# Joint Effect of Habitat Identity and Spatial Distance on Spiders’ Community Similarity in a Fragmented Transition Zone

**DOI:** 10.1371/journal.pone.0168417

**Published:** 2016-12-29

**Authors:** Yoni Gavish, Yaron Ziv

**Affiliations:** Spatial Ecology Lab, Department of Life Sciences, Ben-Gurion University of the Negev, Beer-Sheva, Israel; University of Sydney, AUSTRALIA

## Abstract

Understanding the main processes that affect community similarity have been the focus of much ecological research. However, the relative effects of environmental and spatial aspects in structuring ecological communities is still unresolved and is probably scale-dependent. Here, we examine the effect of habitat identity and spatial distance on fine-grained community similarity within a biogeographic transition zone. We compared four hypotheses: i) habitat identity alone, ii) spatial proximity alone, iii) non-interactive effects of both habitat identity and spatial proximity, and iv) interactive effect of habitat identity and spatial proximity. We explored these hypotheses for spiders in three fragmented landscapes located along the sharp climatic gradient of Southern Judea Lowlands (SJL), Israel. We sampled 14,854 spiders (from 199 species or morphospecies) in 644 samples, taken in 35 patches and stratified to nine different habitats. We calculated the Bray-Curtis similarity between all samples-pairs. We divided the pairwise values to four functional distance categories (same patch, different patches from the same landscape, adjacent landscapes and distant landscapes) and two habitat categories (same or different habitats) and compared them using non-parametric MANOVA. A significant interaction between habitat identity and spatial distance was found, such that the difference in mean similarity between same-habitat pairs and different-habitat pairs decreases with spatial distance. Additionally, community similarity decayed with spatial distance. Furthermore, at all distances, same-habitat pairs had higher similarity than different-habitats pairs. Our results support the fourth hypothesis of interactive effect of habitat identity and spatial proximity. We suggest that the environmental complexity of habitats or increased habitat specificity of species near the edge of their distribution range may explain this pattern. Thus, in transitions zones care should be taken when using habitats as surrogate of community composition in conservation planning since similar habitats in different locations are more likely to support different communities.

## Introduction

Disentangling the effects of niche-based and dispersal-based processes in structuring ecological communities have challenged ecologists for the last few decades [1, 2]. Although both types of processes simultaneously affect community composition [3], their relative effects may have significant consequences to the community’s structure and dynamics. Furthermore, the relative effects of both types of processes may depend on the scale of observation. At small grain sizes, in which individuals of multiple species interact directly with one another, aspects relating to species’ realized niches usually have considerable effect on community composition. The ability of species to cope with interspecific interactions (e.g., competition, predation and facilitation) in a local community involves multiple trade-offs, which are often dependent on the environmental context [4]. The outcome of such interspecific interactions are thus mediated by resource partitioning and habitat preferences [5]. As grain size increases to scales beyond direct interaction of individuals, the relative role of dispersal-based processes in structuring ecological communities increases. That is, the community composition in a focal larger grain size is affected by the ability of individuals of various species to cross dispersal barriers, colonize potential local sites within it, sustain viable populations or maintain non-viable populations through continuous propagule rain [6]. When grain size increase even more, aspects relating to species’ fundamental niches are dominant as species are filtered according to their evolutionary physiological limits [7, 8]. Therefore, at this scale, dispersal limitations will become less important in structuring ecological communities.

As a consequence, both niche-based processes and dispersal-based processes affect the similarity in community composition between two samples (i.e., of a given grain size) taken at a certain spatial distance from one another. In fact, Chase and Myers (2011) [9] suggested that the decay of similarity with environmental and spatial distance can be used to disentangle the effect of deterministic, niche-based processes from those of stochastic, dispersal-based processes. Accordingly, similarity should decay with environmental distance, yet remain constant with spatial distance if community similarity is only affected by aspects of the realized niche. The opposite pattern is expected if community composition is affected only by dispersal-based processes [9]. Therefore, it is important to explore the decay of similarity at small grain-size at which niche-based process are still relevant.

However, exploring the distance-decay of similarity at small-grain sizes is not straight-forward for several reasons. Firstly, Nekola and White (1999) [10] reported that variance in similarity values tend to increase with decreasing grain size, making it difficult to identify weak decay trends when small grain sizes are used. Second, most pairwise similarity values, and other measures of beta diversity are sensitive to alpha diversity values, which usually decrease with decreasing grain sizes. As such, samples taken at small grain sizes are expected to have relatively low basal levels of similarity, regardless of the spatial distance [9, 11]. Finally, empirical studies suggest that the relative role of environment and space also depend on the extent of the study (see S3 Fig in [3]). In fact, simulations in isotropic one-dimensional landscapes suggest that the entire shape of the distance decay curve depends on both grain and extent [12], as well as species aggregation level (as noted also by [11]). Therefore, it is important to explore similarity decay for small grain size, while controlling for local species richness differences, and to do so for various extents.

Here we approach this complexity by moving from continuous spatial and environmental distances to ecologically-defined categories. The categories of the spatial distance can be based on well-defined, natural hierarchical structure in the distribution of natural habitats. For example, two samples can be **a)** from the same patch; **b)** from different patches but from the same landscape (i.e., within the dispersal distances of most individuals); **c)** two samples from adjacent landscapes (connected by long-distance dispersal events); and **d)** two samples from distant landscapes (connected by very rare events of long-distance dispersal). Similarly, environmental distance can be categorized at the local scale according to habitat identity with two samples from the same habitat or from different habitats. Exploring the change in community composition based on such spatial and environmental distance categories may allow testing four general hypotheses:

1. **Habitat identity alone**—if community composition is affected mainly by niche-based processes, pairs of samples from the same habitat will exhibit higher levels of similarity than pairs of samples from different habitats and similarity should not differ considerably between the different distance categories.
2. **Spatial proximity alone**—if community composition is affected mainly by dispersal-based processes, the mean pairwise similarity should decrease from distance category **a** to **d** with no difference between pairs of samples from the same habitat and pairs of samples from different habitats at any distance category.
3. **Non-interactive effect of both habitat identity and spatial proximity**–if community composition is affected independently by niche-based and dispersal-based processes, similarity should decay with distance and be higher at same habitat pairs. However, the difference between the same habitat pairs and different habitat pairs should be similar at all distance categories.
4. **Interactive effect of habitat identity and spatial proximity**–if community composition is affected by both niche-based and dispersal-based processes, yet the two sets of processes interact, similarity should decay with distance and be higher at same habitat pairs. In this case, the difference between same habitat pairs and different habitat pairs should decrease with distance.

**Fig 1:**
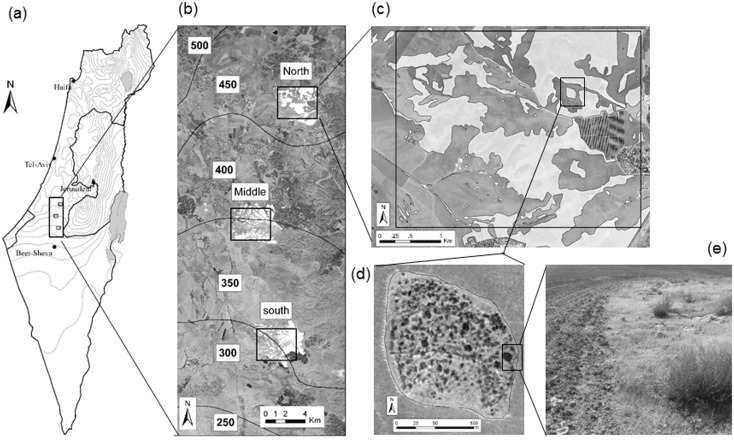
Hierarchical scales of the study system. (a) The sharp precipitation gradient of Israel and location of Southern Judea Lowland (SJL) along the gradient. (b) The north, middle and south landscapes within SJL. Note the sharp decline in mean annual precipitation along a short gradient of 30 km. (c) The north landscape—the distribution of remnant natural patches within the agricultural matrix. (d) A patch, with its specific attributes of area, shape, and internal heterogeneity. (e) One complex habitat (perennial shrub, low right corner) surrounded by a simple habitat (annual plants <15 cm tall), and the contrast with the agricultural matrix during the sampling period.

Despite the simplicity of the above hypotheses, we are not aware of any publications that actually explored them. Thus, in addition to understanding what processes could affect decay similarity in our spider communities, one of the goals of this paper is to produce a simple procedure to disentangle the effects of dispersal-based vs. niche-based processes in scale-dependent systems. More specifically, we explore here the above four hypotheses using fine-scale samples of spiders along a 30 km sharp climatic gradient in Southern Judea Lowlands (SJL), Israel (Fig 1a). SJL offers a unique opportunity for studying the effects of scale-dependent processes on species diversity and community structure (e.g. [13–15]). At the regional-scale, SJL reflects a sharp climatic gradient, in which mean annual precipitation drops from 450 mm/year in the north to 250 mm/year in the south [16], without any considerable change in elevation, geological and lithological conditions (Fig 1b). The steep drop of mean annual precipitation over this short distance results with a considerable change in plant biomass [17] and floral community composition [13, 18, 19]. Faunal communities also change considerably along this gradient, with Mediterranean-oriented species being more dominant in the north and arid-oriented species in the south [20]. At the landscape scale, SJL is highly fragmented (Fig 1C), and most natural flora and fauna are found in remnant natural patches [13, 21, 22], embedded within the agricultural matrix. The natural patches (Fig 1d) differ from one another in their area, shape, internal heterogeneity, and spatial context. Finally, at the very local scale, SJL contains several well-defined habitats that differ from one-another in various aspects such as structural complexity, plant biomass, microclimatic conditions and prey availability.

## Methods

### Sampling

We focused our sampling in SJL, Israel (Fig 1b, 31°23'60''-31°40'50'' N, 34°47'50–34°52'30' E), located within the steep climatic transition area of Israel (Fig 1a). We used three 3.2×4 km landscapes, in the south (Dvir), middle (Lachish) and north (Galon) of SJL. In all three landscapes (Fig 1b), remnant patches of natural vegetation (Fig 1c and 1d), characterized by different area, shape, internal heterogeneity and spatial context, are embedded in a matrix of agricultural fields, mainly wheat. The wheat starts growing at the mid rainy season (January) and is harvested in May-June. Thereafter, the remaining straw is collected and the fields are kept open and bare until the next growing season. We sampled vegetation and spiders between June and early September in 2007, when the bare fields were characterized by exposed soil. Most spider species cannot complete their lifecycle within the fields when it is covered by exposed soil, and must use the natural habitat for their survival (see [22]).

We sampled 13 patches in each of the south and middle landscapes and 9 patches in the north landscape. Sampling was stratified according to nine a-priori defined habitats (see an example in Fig 1e). The nine habitats included: exposed soil, annual plants <15 cm tall, annual plants ≥15 cm tall, rosette plants (mainly *Asphodelus ramosus*), *Sarcopoterium spinosum*, *Hyparrhenia hirta*, shrubs (<35 cm tall), bushes (≥35 cm tall), and thistles (mainly *Silybum marianum* and *Notobasis syriaca*). The nine habitats can be divided to two main groups that differ considerably in various environmental aspects. The first group is more environmentally simple and contain the first four habitats. The second group is more environmentally complex and contain the remaining five habitats. Among others, the group of complex habitats are more structurally complex, have considerably higher plant biomass, have higher abundance and diversity of potential prey and provide more shade in the hot days of the summer, when temperatures can rise to 45°Celsius. This separation was based on close knowledge of our system and clear familiarity with the focal group of organisms. However, we also confirmed this separation by explicitly analysing and comparing the different habitats (see below).

We focus on spiders due to their high species richness [23], their high abundance in arid and semi-arid environment (see citations within [24]), and their potential role as bioindicators for other arthropod groups [25]. In addition, spiders exhibit profound habitat selection behaviour, resulting in strong correlations between habitat structure and community structure [26–29]. Finally, spider exhibit two main modes of dispersal—a relatively long distance ballooning dispersal and a more local cursorial dispersal—that differ both in distance and in sensitivity to matrix structure [30, 31].

To sample spiders we used a stratified random sampling scheme. Each 0.5×0.5 m quadrat was located at least 5 m from the edge of the patch (to avoid an edge effect) and contained a single habitat. We ensured sampling at least one quadrat from each complex habitat we observed in each patch, because preliminary sampling revealed that spider abundance and species richness in the complex habitats was significantly higher than in the simple ones. Each patch contained ≥7 quadrats and sampling effort increased with patch size. A total of 216, 230 and 198 samples containing spiders were taken in the south, middle and north landscapes, respectively. Additional 57 samples (all from structurally simple habitats) did not contain any spiders and were excluded from all analyses.

To obtain samples, we used the vacuum option of a leaf blower with a mesh (0.5 mm) sleeve inserted within the suction tube [32]. For annual plants and exposed soil, we repeatedly placed the suction tube above the quadrat for one minute. For perennials, during the one-minute sample we first placed the suction tube above the external parts of the plants and then inserted the suction tube into the internal sections of the plant and into the debris under the plant. All the sucked content was taken to the lab. All spiders >0.5 mm in total length were separated from debris with a set of sieves and preserved in 70% ethanol. Spiders were identified to the lowest possible taxonomic level. If we could not determine the species of an individual, we classified it as a morphospecies [22]. Sampling arthropods in these locations does not require any specific permissions. We confirm that the field studies did not involve endangered or protected species.

### Data preparation and analysis

First, we square-root transformed the abundance data of each species in each sample to reduce the importance of dominant species in the similarity calculations [33]. We then calculated Bray-Curtis similarity between all sample-pairs and used the resulting similarity matrix throughout the analyses. To explore if indeed our grouping of the nine habitats to four simple and five complex ones is valid, we conducted a non-metric multidimensional scaling (nMDS) analysis based on all pairwise similarity values. For the nMDS analysis we used the *metaMDS* function in the *vegan* package [34] in R [35]. We further compared the mean number of species per sample and the mean number of individual per sample in simple and complex habitats using t-test.

Next, we divided the similarities between two samples to four distance categories in accordance with the above four general hypotheses: 1. Two samples from the same patch (within-patch); 2. Two samples from different patches but from the same landscape (between-patches); 3. Two samples from adjacent landscapes—i.e., south-middle or middle-north; 4. Two samples from distant landscape—i.e., one from the south and one from the north landscapes. The distance categories correspond to median distances of 441 (lower-upper quartiles: 78–1,001), 1,508 (1,026–2,044), 12,760 (12,060–13,380) and 24,630 (24,030–25,350) m for within-patch, between-patches, adjacent landscapes and distant landscapes, respectively. In each distance category we then divided the sample-pairs into two habitats categories—samples from the same habitat or from different habitats.

We used a two-way non-parametric MANOVA to explore the effect of distance, habitat and the interaction between them [36]. We first calculated F-values according to observed values for the two main effects—distance and habitat—and the interaction between them. We then randomly permutated observed similarity values 999 times, and calculated F-values for each permutation. Significance values (pseudo-p) were calculated as the proportion of permutation that had equal or larger F-values than the observed one [36]. For a post-hoc test, we used a similar one-way MANOVA and permutation for comparing the same and different habitats, for each distance category separately. In addition, we used a similar one-way non-parametric MANOVA to compare any two-distance values for the same or different habitats categories (but not same vs. different habitats at different distances).

We ran this analysis for all pairs of samples. However, we have repeated the analyses three additional times while further classifying samples as those from simple habitats and complex habitats. We did so since complex habitats contains considerably higher spider diversity and abundance, as well as higher prey diversity and abundance such that stratifying the analysis according to habitat complexity may reveal the effect of local diversity and abundance on the emergent pattern. If local species assemblage is a random subset of the species pool, two samples containing a large subset each are more likely to share species than two samples with a small subset. The effect will be even stronger if species are not chosen randomly, but based on species habitat preferences. Therefore, we predict that the overall levels of similarity will be higher for pairs of samples from two complex habitats relative to two samples from two simple habitats. We further predict that the emergent pattern will be clearer for complex habitats than for simple ones, due to less variation in pairwise similarity values. Therefore, in total we repeated the analysis four times, using: 1) pairs of all samples; 2) pairs in which both samples were taken in any of the four simple habitats; 3) pairs in which both samples were taken in any of the five complex habitats; 4) pairs in which one sample is from one of the four simple habitats and one sample is from one of the five complex habitats. For the last analysis, the same habitats pairs were the same as in the first analysis- i.e., all pairs in which both samples were taken in the same habitat, regardless of their complexity. All analyses were conducted in R [35].

## Results

### General results

**Fig 2:**
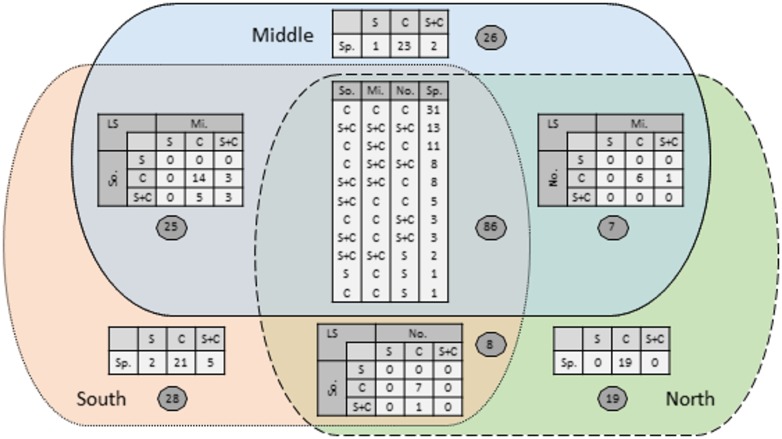
Species distribution in the three landscapes. Venn diagram indicating the number of species (Sp.) found exclusively in the south (So.), middle (Mi.) and north (No.) landscapes (LS), shared by any two landscapes, and shared by all three landscapes. In each area, the embedded tables indicate the species that occurred only in simple habitats (S), only on complex habitat (C) or in both simple and complex habitats (S+C).

All data required to repeat the analyses carried here can be found in S1 Table and S1 File. The 644 samples resulted with a total of 14,854 spider individuals, from 32 families and 199 species or morphospecies. From the 199 species, 86 occurred in all three landscapes, 25, 7 and 8 species occurred in both the south and middle, middle and north and south and north landscapes, respectively (Fig 2). The south, middle and north landscapes contained 28, 26 and 19 unique species, respectively (Fig 2).

### Difference between simple and complex habitats

**Fig 3:**
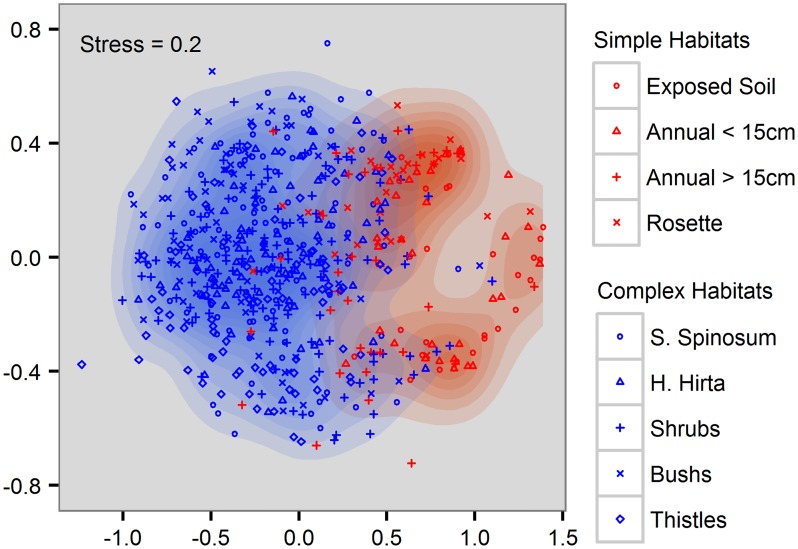
non-metric multidimensional scaling of all samples. Note the clear separation between the simple habitats and the complex habitats along the first axis. The second axis corresponded mainly to landscape (S1 Fig).

In all three landscapes, the average number of individuals per-sample was significantly lower in the simple habitats than in the complex habitats (t test, p<0.001, Table 1). Similar results were found for the average number of species per-sample (t test, p<0.001, Table 1). The difference between complex and simple habitats would increase even more, had we included the 57 samples with no spiders, all taken in simple habitats. From the 199 species, 61% appeared only in complex habitats, 38% in both simple and complex habitats and only 1% appeared only in simple habitats (the simple habitats accounted for 34% of the samples). Similar results were obtained when exploring each landscape separately. The two groups of habitats where highly separated in the nMDS analysis (Fig 3). The five complex habitats formed a single cluster with no clear separation according to habitat. However, complex habitat samples were relatively separated according to landscape along the second nMDS axis (S1 Fig). In contrast, the four simple habitats formed three clusters, which do not correspond in a simple manner to neither habitats nor landscapes.

**Table 1:** The average (± s.e.) number of individuals and species per sample in structurally simple and structurally complex habitats, for the three landscapes.

Measure	Habitat	South	Middle	North
Abundance	Simple	4.21 (0.65)	6.05 (0.92)	3.45 (0.37)
	Complex	23.68 (1.39)	41.50 (2.51)	21.50 (1.25)
Species	Simple	2.57 (0.22)	3.46 (0.37)	2.43 (0.21)
	Complex	9.81 (0.33)	12.85 (0.47)	9.59 (0.35)

### The four hypotheses

**Fig 4:**
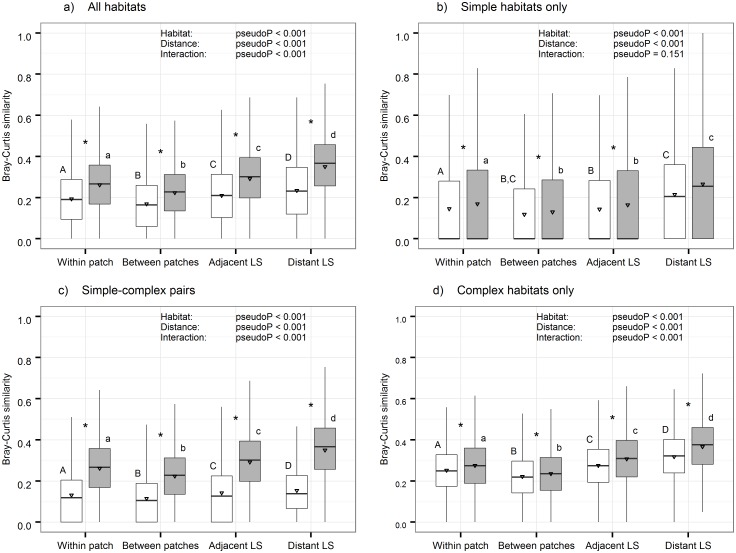
Results of the two-way non parametric MANOVA analyses. Median, 25 and 75 percentiles (± 1.5 inter quantile range) and average (triangle) similarity in spider community structure between pairs of samples. Pairs of samples are divided to 4 distance categories and 2 habitats categories—same habitat (white) or different habitats (grey). The panels represent four stratifications: (a) all habitats, (b) two samples from simple habitats, (c) one sample from a simple habitat and one from a complex habitat (d) two samples from complex habitat. In each panel, results of two-way non-parametric MANOVA are given. Distance categories that did not differ in the post-hoc are labelled with a similar capital letters (same habitat) or lower-case letters (different habitats). Within a distance category, significant differences between same habitat pairs and different habitats pairs are given as *.

The two-way non-parametric MANOVA indicated a significant effect of habitat and distance, as well as a significant interaction term in three of the four analyses (Fig 4). In these three analyses (all habitats, simple-complex pairs and complex habitat only: Fig 4a, 4c and 4d, respectively), we also found all post-hoc tests to be statistically significant. That is, in each distance category the average similarities between pairs of samples from the same habitat were higher than between pairs of samples from different habitats.

Similarly, all distance categories differed significantly one from the other, indicating a gradual change in species composition along the climatic gradient. In the simple-simple habitats pairs, no significant interaction term was found, yet a significant effect of habitat identity and spatial distance was observed (Fig 4b). In this analysis we found that similarity dropped as soon as two samples from different patches were taken, even if they were from the same landscape, and did not further decline considerably with distance.

Fig 4 presents several other important results. First, the complex habitat pairs (Fig 4d) showed higher average level of similarity than any combination with samples from a simple habitat (Fig 4b and 4c). Second, in the simple-simple habitat combinations (Fig 4b), in all, but the within patch distance category, the medians were zero, indicating that most pairs of samples did not share any species. Third, the difference between same habitat pairs and different habitat pairs was highest in the simple-complex pairs (Fig 4c), probably since simple habitats and complex habitat differ considerably in alpha diversity. Similarly, the difference between same habitat pairs and different habitat pairs was smallest in the complex-complex pairs (Fig 4d), as the samples contained relatively high species richness with relatively low levels of habitat specificity.

## Discussion

Classic ecological theory posits that fine-scale community composition is mainly affected by niche-based processes that relate to direct interactions between individuals/species. However, the community at any given location is shaped by the structure of the community at larger spatial scales. In SJL, the community composition at any given sample is affected by the habitat in which the sample was taken, as evident by the effect of habitat identity on community similarity (Figs 3 and 4). However, the community composition at the patch scale constrains the local community, since each habitat can only support a subset of the species that occurs in the patch. At the patch scale, community composition is affected by habitat heterogeneity, disturbance and the spatial context of the patch (YG, unpublished results). Yet it is also constrained by the landscape-scale community, as most of the individuals that disperse to any patch arrive from other patches close by. Similarly, the community composition at any landscape is likely affected by the metapopulation dynamics of multiple species and by the penetrability of the agricultural matrix. However, each landscape differs from other landscapes in the species that are filtered out from the regional species pool. The unique location of SJL along a very sharp climatic gradient and at the boundary of several biomes suggests that the three landscapes may actually contain species from different biogeographic origins. The clear results also demonstrate that the SJL serves as an excellent model system to study such scale-dependent patterns and processes.

Despite this complexity of interacting processes, we used stratification according to habitat identity to explore four main hypotheses—that community composition is affected by i) Habitat identity alone; ii) Spatial proximity alone; iii) Non-interactive effect of both habitat identity and spatial proximity; iv) Interactive effect of habitat identity and spatial proximity. In accordance with other cases [3], we found that both niche-based processes and dispersal–based processes affect community composition, ruling out our first two hypotheses. We further found a significant interaction between habitat identity and spatial distance, such that the difference in mean similarity between same habitat pairs and different habitats pairs decreases with distance (Fig 4a).

The decay of similarity with distance has been previously explored for spiders (e.g., [37, 38]). Despite their small sizes, spiders are usually considered as good dispersers, as evident by their role in colonization of newly-formed volcanic islands. Such long-distance dispersal is achieved through a ballooning behaviour, which is independent from the matrix configuration or identity. However, in fragmented landscapes, spiders have been shown to reduce ballooning tendency [39]. Additionally, this form of dispersal is not employed by all spider families. Although accurate data on cursorial dispersal distance is scarce, mark-recapture studies usually do not reveal distances of more than few meters [40, 41], yet daily foraging distances of up to 200 meters are reported (see details in [25]). Spiders increase their mobility during two periods of their life cycle—the transition to solitary life by a juvenile and the reproduction period in which adult male search for females [25]. We are unaware of records of distance travelled in these periods, yet most spiders probably do not exceed hundreds of meters. Therefore, even in the lack of a strong environmental gradient we expected spider similarity to decay with distance due to dispersal limitations.

Similarly, the strong effects of habitat identity on spider composition similarity at small distances is not surprising, given spiders’ sensitivity to habitats’ structural complexity, prey availability and microclimate conditions [27, 29, 42–44]. Indeed, all these aspects are likely to affect the observed differentiation between simple and complex habitats (Fig 3). However, the effect of habitat identity at larger distances is not straightforward. SJL lies at the meeting points of four phytogeographical regions—Mediterranean, Irano-turani, Saharo-Sindi and Sudanese-Dekken [45]—and contains considerable mixture of two zoogeographic realms: the Saharo-Arabian and Palearctic [46]. Although the biogeographic origins of spiders are not yet resolved, we expected considerable spider species turnover between the three landscapes. If turnover between landscapes is high, we would expect that the same habitat pairs and different habitats pairs at the largest distance category to be quite similar to one another, since the differences in species pool constrain the species found in each sample. From the 199 species we recorded, 86 (43.2%) were found in all three landscapes, 40 (20.1%) in only two landscapes, and 73 (36.7%) in a single landscape (Fig 2). Similar proportions of shared species were also reported in studies of plants and reptiles conducted at the same three landscapes. For plants [13], a total of 428 species were recorded when sampling at the same three landscapes, from which 192 (44.9%) were found in three landscapes, 97 (22.7%) in two landscapes and 139 (32.4%) in a single landscape. For reptiles [20], a total of 29 species were recorded in the three landscapes, from which 12 (41.4%) were found in all three landscapes, 8 (27.6%) in two landscapes and 9 (31.0%) in a single landscape. Both these studies showed that much of the turnover is based on species biogeographic origin.

However, our analysis revealed significant differences between same and different habitat pairs even at the largest distance category. One explanation is that the difference for the distant landscapes category in Fig 4a is affected by low similarity values of two samples from different habitats, if the two habitats differ considerably in species richness and abundance (Table 1). Indeed, when controlling for local richness and abundance, the largest difference at this distance category was observed in simple-complex pairs (Fig 4c), while in two complex habitats pairs the difference was very small (yet significant, Fig 4d). For two simple habitats the difference between the means was relatively small as well, yet the medians showed higher variation due to many zero similarities. In fact, the many zero similarities of simple-simple pairs arise since most species shared by two or more landscapes are found exclusively in structurally complex habitats (Fig 2). For example, from the 86 species shared by all landscape, 31 are only found in complex habitats in all three landscapes, and only four species are found only in simple habitat in at least one landscape (Fig 2). Overall, these results indicate that accounting for alpha-diversity values by habitat stratification may be used to identify weak signals of distance decay. They further suggest that clearer similarity decay patterns are likely to be observed when focusing on fine scale locations that contain species rich habitats.

Another explanation for the observed difference at large distance categories is that, despite a relatively high turnover rate, still a large enough number of species were found in all three landscapes, and that for these species the entire SJL lies at the edge of their global distribution pattern. In fact, the 86 spider species found in all three landscapes represent 58%, 60% and 72% of the total species recorded in the south, middle, and north landscapes, respectively. Species near the edge of their distribution range are expected to become more specific in their habitat requirements [47]. Empirically, it has been shown for diverse groups, including epiphytes, trees and butterflies [47–49]. At least partially, the higher habitat specificity may be explained by changes in within-habitat microclimatic conditions along macroclimatic gradients [50]. Therefore, a species whose core distribution is at the Mediterranean ecosystem becomes restricted to only a few suitable habitats in SJL, and as such increases the mean similarity for same-habitat pairs. From the other direction of the gradient, a species highly adapted to the desert condition in the south of Israel (and further into the Saharo-Arabian realm), will exhibit habitat preferences for other habitats, and again will increase the same-habitat’s mean similarity and decrease the different-habitats’ mean similarity. If this is so, further increasing the extent of this study to the north and south eventually should lead to non-significant difference between same-habitat and different-habitats pairs. At this scale, habitat identity should better not be used as surrogate for community composition, as assumed in many conservation planning.

The relative effect of spatial proximity and habitat identity was examined in the North American transition zone between shortgrass-steppe and Chihuahuan desert for several arthropod taxa, including ants [51], crickets/grasshoppers and spiders [52]. For all those taxa, the spatial proximity had a more potent effect on community similarity than habitat identity. However, the results presented here suggest that the relative influence of spatial proximity and habitat identify are distance-dependent, which had not been considered for the North-American studies.

We argue that habitat identity may countervail the effect of distance, as long as the species pool is relatively homogeneous. In transition zones between biogeographic species pools, such as the SJL and the North American shortgrass-steppe to desert transition zone [51, 52], the ability of habitat identity to countervail the effect of distance depends upon the degree of change of the gradient. In Bestelmeyer and Weins (2001) [51] the change in precipitation from 320 to 232 mm per-year occurs over distance of approximately 600 km. In Lightfoot et al. (2008) [52], the additional altitude at the north-most site results with a drop in precipitation from approximately 475 to 225 over 300 km. The gradient in our system (SJL) is very sharp relative to the North American transition zone. Therefore in a distance of about 25 km the difference between similar habitat pairs and different habitat pairs in community similarity is already very small (e.g., Fig 4d). In all three cases the gradient represents a change in the dominant species pool along a transition zone.

As it is practically impossible to tailor-suit a conservation plan for every species, habitats are commonly used as surrogates in conservation planning. By knowing the community composition of different habitats, alternative conservation plans can be compared based on the complementarity of the species conserved [53]. Our results suggest that such approaches may indeed be feasible when the regional species pool is relatively homogeneous. However, in transitions zones, habitats at different locations may support different communities, and as such may be better treated as different entities in any complementarity analysis. Hopefully, analyses similar to the one conducted here may shed lights on habitats-dependent similarity decay rates, and help identify better conservation plans.

## Supporting Information

S1 Fig The results from the nMDS analysis, with emphasis on the landscape origin of the samples. (DOCX)

S1 File metadata information for S1 Table. (DOCX)

S1 Table The number of individuals from each species or morphospecies in each sample, alongside the landscape, patch code, habitat and structural complexity of each sample, and coordinates. (CSV)
