# Correction: Joint Effect of Habitat Identity and Spatial Distance on Spiders' Community Similarity in a Fragmented Transition Zone

**DOI:** 10.1371/journal.pone.0173326

**Published:** 2017-02-28

**Authors:** Yoni Gavish, Yaron Ziv

In Fig 4, the order of the labels “Within patch”, “Between patches”, “Adjacent LS”, and “Distant LS” on the x-axis of the box-and-whisker plots is incorrect. The grey and white shading of the bars is also incorrectly switched. Please see the corrected Fig 4 here.

**Fig 4:**
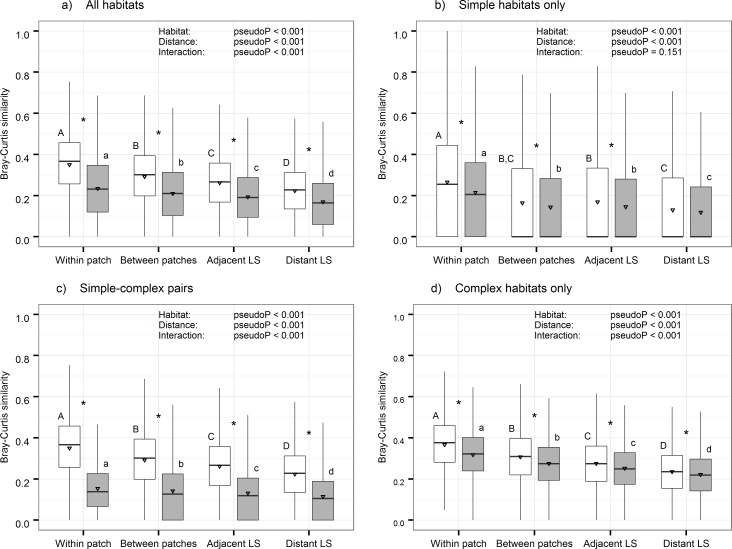
Results of the two-way non parametric MANOVA analyses. Median, 25 and 75 percentiles (± 1.5 inter quantile range) and average (triangle) similarity in spider community structure between pairs of samples. Pairs of samples are divided to 4 distance categories and 2 habitats categories—same habitat (white) or different habitats (grey). The panels represent four stratifications: (a) all habitats, (b) two samples from simple habitats, (c) one sample from a simple habitat and one from a complex habitat (d) two samples from complex habitat. In each panel, results of two-way non-parametric MANOVA are given. Distance categories that did not differ in the post-hoc are labelled with a similar capital letters (same habitat) or lower-case letters (different habitats). Within a distance category, significant differences between same habitat pairs and different habitats pairs are given as *.
