# Supplementary material for: Joint Effect of Habitat Identity and Spatial Distance on Spiders’ Community Similarity in a Fragmented Transition Zone
Source: PLoS One. 2016 Dec 29;11(12):e0168417. doi: 10.1371/journal.pone.0168417 (PMC5199073; doi:10.1371/journal.pone.0168417)

Title: Joint effect of habitat identity and spatial distance on spiders’ community
similarity in a fragmented transition zone

Authors: Gavish, Yoni [gavishyoni@gmail.com](mailto:gavishyoni@gmail.com)

Ziv, Yaron yziv@bgu.ac.il

S1 Fig. **The results from the non-metric multidimentional scaling (nMDS )analysis of all pairwise similarity values.** The figure exhibits the same results as Fig 3 in the main text, only here the point’s colour represents the landscape in which the samples were taken, while the points’ shape separates between simple and complex habitats. Not the clear separation of the three landscape along the second axis.


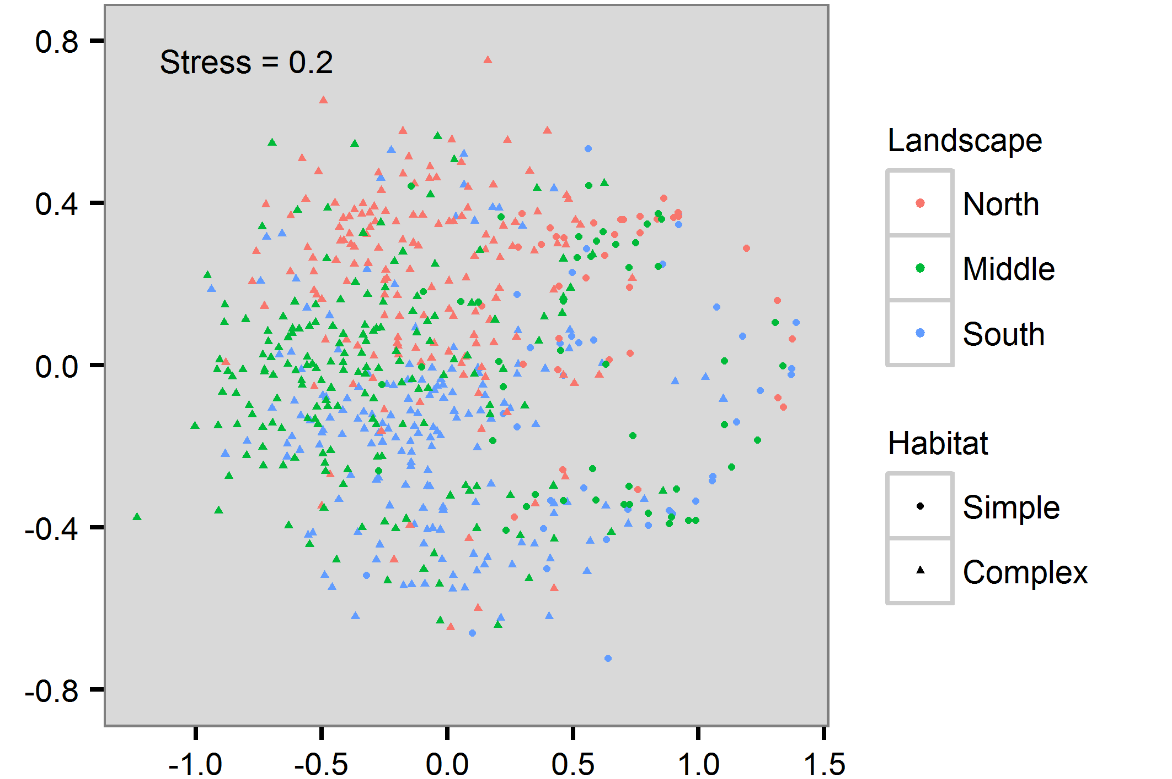

Supplement: S1 Fig — (DOCX) [file pone.0168417.s001.docx]
