# Supplementary material for: Joint Effect of Habitat Identity and Spatial Distance on Spiders’ Community Similarity in a Fragmented Transition Zone
Source: PLoS One. 2016 Dec 29;11(12):e0168417. doi: 10.1371/journal.pone.0168417 (PMC5199073; doi:10.1371/journal.pone.0168417)
Supplement: S1 File — (DOCX) [file pone.0168417.s002.docx]

Title: Joint effect of habitat identity and spatial distance on spiders’ community
similarity in a fragmented transition zone

Authors: Gavish, Yoni [gavishyoni@gmail.com](mailto:gavishyoni@gmail.com)

Ziv, Yaron yziv@bgu.ac.il

**MetaData information on the S1_Table.csv**

The S1_Table.csv file contains the abundance of each spider species or morpho-species in each of the 643 samples used in the analysis alongside additional information on each sample.

Table S1: column names and their meaning

| Name | Value |
| --- | --- |
| Landscape | The landscape in which the sample was taken. Either: Dvir, Lachish or Galon, corresponding to the south, middle and north landscapes in the main text. |
| PatchCode | A unique ID for each patch. |
| sample | A unique ID for each sample. |
| Habitat | The habitat of each sample: |
| SimCom | Is the habitat structurally simple (Sim) or complex (Com) |
| x | The X coordinate of the sample in Israel new TM coordinate system |
| y | The Y coordinate of the sample in Israel new TM coordinate system |
| All other columns | Species names, first word is the spider family |

Table S2: Habitat categories and their structural complexity

|  | Name | Main Text | Structural complexity |
| --- | --- | --- | --- |
| 1 | ExposedSoil | exposed soil | Simple |
| 2 | Annual<15cm | annual plants <15 cm tall | Simple |
| 3 | Annual>15cm | annual plants ≥15 cm tall | Simple |
| 4 | Rosette | rosette plants (mainly *Asphodelus ramosus*) | Simple |
| 5 | SarcopoteriumSpinosum | *Sarcopoterium spinosum* | *Complex* |
| 6 | HyparrheniaHirta | *Hyparrhenia hirta* | *Complex* |
| 7 | Shrub | shrubs (<35 cm tall) | *Complex* |
| 8 | Bush | bushes (≥35 cm tall) | *Complex* |
| 9 | Thistles | thistles (mainly *Silybum marianum* and *Notobasis syriaca*) | *Complex* |
